# Essential role of reliable reduction quality in internal fixation of femoral neck fractures in the non-elderly patients—a propensity score matching analysis

**DOI:** 10.1186/s12891-022-05307-8

**Published:** 2022-04-11

**Authors:** Longhai Qiu, Yuliang Huang, Guowen Li, Hongbo Wu, Yu Zhang, Zhiwen Zhang

**Affiliations:** 1 Department of Traumatology and Orthopaedic Surgery, Orthopaedic Institute, Huizhou Municipal Central Hospital, Huizhou, 516001 China; 2 The Second School of Clinical Medicine, Southern Medical University, Guangzhou, 510515 China; 3 Department of Orthopedics, Guangdong Provincial People’s Hospital, Guangdong Academic of Medical Science, Guangzhou, 510080 China

**Keywords:** Femoral neck fractures, Internal fixation, Necrosis of femoral head, Arthroplasty, Reoperation

## Abstract

**Background:**

The rate of failure of internal fixation for femoral neck fractures has remained largely unchanged over the past 30 years. The current study attempted to identify the controllable variables influencing the failure of internal fixation of femoral neck fractures.

**Methods:**

The study included 190 patients aged from 20 to 65 with femoral neck fracture caused by low energy violent injuries (fall from standing height), who were treated with multiple cannulated screws over the period 2005–2019 at a single centre. Kaplan–Meier (KM) survival analysis was firstly utilized to evaluate the potential interaction between each variable and cumulative rates of reoperation. If *P* < 0.1 in KM survival analysis, the variables would be included in subsequent Cox survival analysis to explore the influencing need for reoperation of a femoral neck fracture. Next, all of the 190 patients were divided into perfect reduction group (Garden Alignment Index I) and imperfect reduction group (Garden Alignment Index II, III, IV). Propensity score matching (PSM) analysis resulted in 39 pairs. After the baseline variables were balanced between the two groups, cox survival analysis was utilized again to explore the variables influencing the need of reoperation of a femoral neck fracture. Finally, KM survival analysis was utilized to compare the cumulative rate of reoperation between perfect reduction (Group PR) and imperfect reduction (Group IR) as a subgroup analysis.

**Results:**

Before PSM analysis, the mean age was 49.96 ± 12.02 years and the total reoperation rate was 17.40%. Cox survival analysis showed that only reduction quality was interrelated with the need for reoperation before PSM analysis and after PSM analysis. Kaplan–Meier cumulative reoperation rate was higher in Group IR than in Group PR after PSM analysis.

**Conclusion:**

To prolong the service life of the original femoral head, it is essential to achieve a completely anatomical reduction and maintain the reduction quality until the patient fully recovers.

## Background

Worldwide, a total of 4.5 million people per year become disabled after a hip fracture. It can be estimated that the number of people living with disability due to hip fracture will increase to 21 million in the next 40 years [1]. As is well-known, artificial femoral head arthroplasty or total hip arthroplasty is the preferred treatment for the elderly with femoral neck fractures, especially for those over 65 years of age [2]. However, preserving the femur head is the primary option for the non-elderly with femoral neck fractures owing to the limited working life of an artificial hip prosthesis [3, 4]. For these patients, the popular processing scheme is a closed reduction followed by internal fixation (CRIF) [5, 6]. Open reduction and internal fixation (ORIF) is applied if the closed reduction failed. Options for internal fixation consist of multiple cannulated screws, a dynamic hip screw (DHS) or a proximal femoral locking plate. Multiple cannulated screws is the classical internal fixation treatment [2]. Femoral head necrosis and fracture nonunion have always been the two major complications of femoral neck fracture treatment, which greatly increases the difficulty of treatment and places a high burden on social and medical resources. The final treatment outcome of these two complications is conversion surgery to a revision or hip replacement surgery (such as hemiarthroplasty or total hip arthroplasty).

As previous studies have demonstrated, the reoperation rate for a failure of internal fixation ranges from 10% to 48.8% and has remained largely unchanged over the past 30 years [7, 8]. Usually, failure is thought to interrelate with the severity of the fracture type, shear force strength, the interval between injury and primary operation, age and the reduction quality. In previous studies, different influencing factors led to statistical variability and randomized controlled study could avoid various uncertain factors of retrospective study. However, a randomized study is difficult to implement due to the unpredictability of trauma.

Therefore, we adopted propensity score matching analysis (PSM), a statistical analysis method, to eliminate the unbalanced baseline variables [9]. To our knowledge, this is the first retrospective study applying PSM to analyze the potential variables of reoperation of internal fixation in the non-elderly with femoral neck fracture. Before beginning the study, we hypothesized that femoral neck fracture fixation failure is related to fracture type severity, the interval between the injury and primary surgery, the stability of fracture and the accuracy of reduction. Previous studies have also pointed out that anatomical reduction is helpful to reduce the shear stress of femoral neck fracture, so as to reduce the use of implants. We set this as an extremely important factor.

## Methods

This report describes a retrospective single-center case–control trial including active patients with femoral neck fractures treated with multiple cannulated screws. Ethical approval was obtained from the Institutional Review Board of Huizhou Municipal Central Hospital, and the study conformed to the tenets of the Declaration of Helsinki. We obtained the verbal consent of the patient or his directly-related family members.

### Patients

**Fig. 1:**
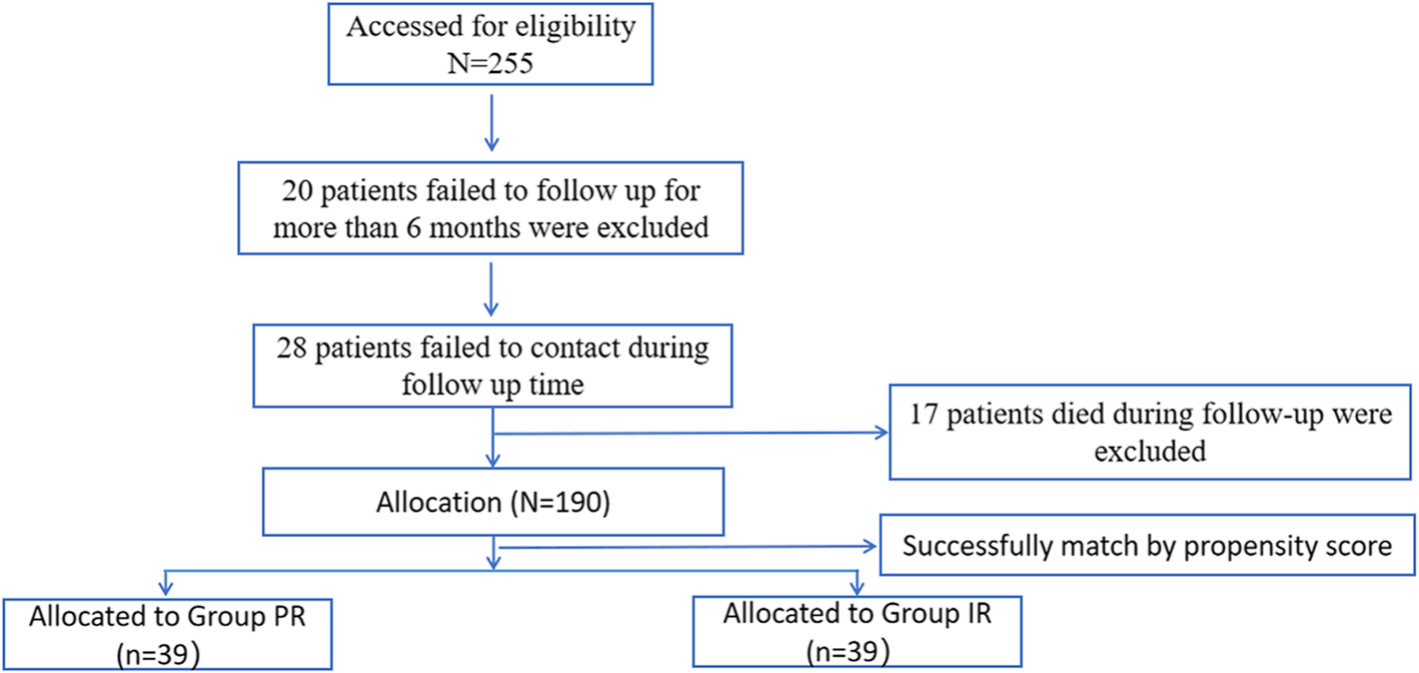
Flow of the patients through each stage of the study

Demographic and procedural data were collected from hospital charts or the database. Follow-up was achieved by using a telephone questionnaire directly or through a visit to the outpatient clinic of the Department of Traumatology and Orthopedic Surgery, Huizhou Municipal Central Hospital. A total of 255 cases of femoral neck fractures were treated by internal fixation with cannulated screws between November 2005 and November 2019. The patient inclusion criteria were: (1) aged between 20 and 65 treated with multiple cannulated screws; (2) where there was no abnormality of lower limb function before the injury;(3) the patient systematically returned to the hospital for review, X-rays would be performed regularly in the 1st, 3rd, 6th and 12th month after operation. Patient exclusion criteria were: (1) those who had bone promoting materials or angiogenic materials used during the surgical procedure; (2) where the participating surgeons had performed fewer than 25 hip fracture fixation procedures during their career; (3) pathological fractures or skeletally immature patients; (4) fractures in patients with pre-existing significant hip arthritis or hip fractures; (5) where the patients had severe multiple injuries that affected their postsurgical functional training, such as severe head trauma, organ damage, or multiple injuries throughout the body; (6) where the follow-up time was less than 6 months; (7) the patient died or could not be contacted when follow-up was sought for the study. Of the 255 patients, 20 patients with less than 6 months follow-up, 28 patients could not be contacted and 17 patients died during follow-up were excluded. Data from the remaining 190 patients were analyzed (Fig. 1).The follow-up rate was 74.51%.

### Variables

**Fig. 2:**
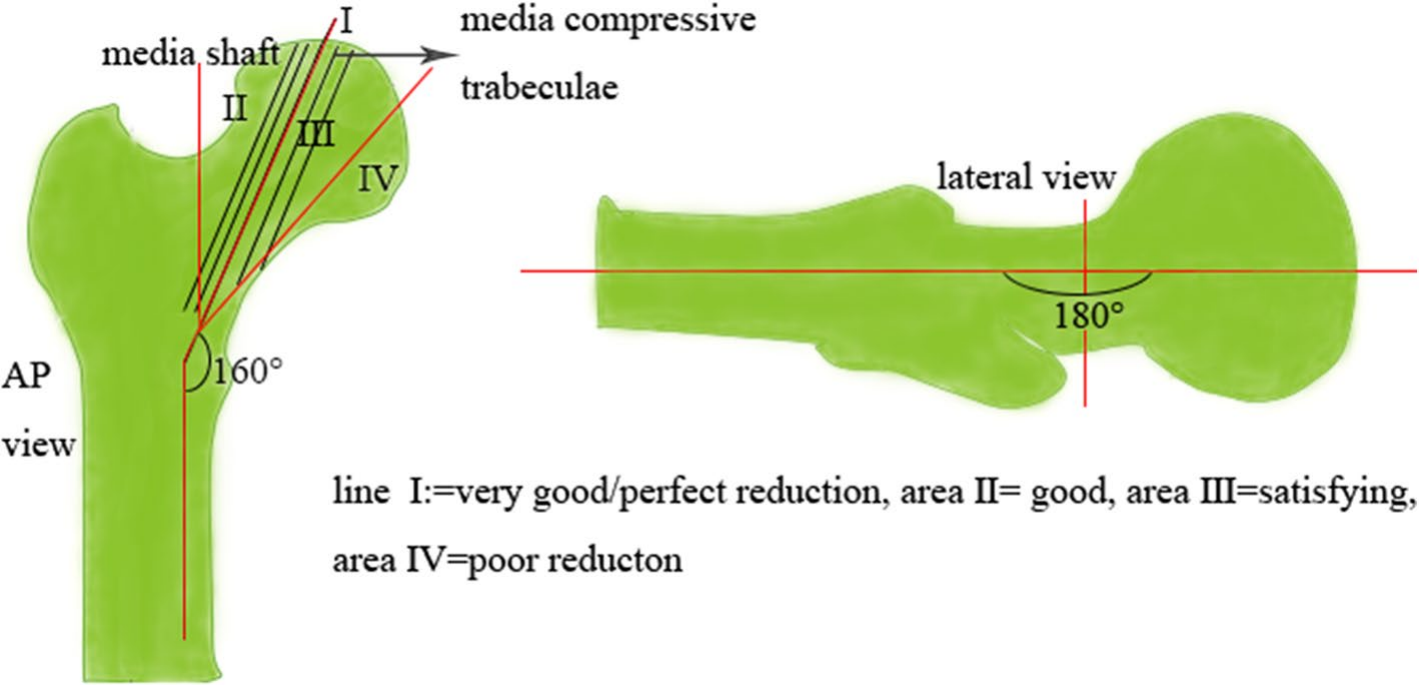
On the AP image, the angle between the medial shaft and the central axis of the medial compressive trabeculae should measure between 160 and 180 degrees. Line I = perfect reduction. Area II = good. Area III = satisfying. Area IV = poor reduction

The following clinical variables were examined after retrospectively extracting them from the patients’ medical records: body mass index (BMI), sex, smoking, injured side, diabetes, occupation (heavy manual worker or not), interval between the injury and surgery (less than 72 h or over 72 h), reduction quality (Garden Alignment Index), location of fracture line (subcapital fracture, transcervical fracture, substrate fracture), Garden Classification (I, II, III, IV), Pauwels Classification (I, II, III), age and follow-up time. Garden alignment index is a standard to judge the quality of fracture reduction [10]. In this study, we selected and assessed the X-rays of the final follow-up as Garden Alignment index (Fig. 2), which was documented by two co-first authors and disagreement was resolved by negotiation if necessary. We divided the Garden Alignment Index into two groups: Group PR (perfect reduction:Garden Alignment index I) and Group IR (imperfect reduction: Garden Alignment index: II, III, IV). The endpoint of maintaining the femur head was defined as a reoperation program that aimed to improve hip function, such as hemiarthroplasty (HA), total hip arthroplasty (THA) and revision surgery. The survival time of femur head was defined as the interval between the primary operation and the need for reoperation due to complications.

### Statistical analysis

#### Interaction between variables and reoperation before PSM analysis

The univariate analysis steps were as follows: the interaction between each measurement data and reoperation was analyzed with Kaplan–Meier (KM) survival analysis one by one; then, the interaction between each continuous variable and reoperation was analyzed with Cox proportional-hazards models one by one (Table 1). If the *P* value was less than 0.1, this group of variables would be included in the subsequent Cox analysis as a suspicious influencing variable of reoperation. A Cox proportional-hazards model was constructed to evaluate the hazard ratio for each event and reoperation.

**Table 1:** Baseline variables between non-perfect reduction and perfect reduction before PSM and after PSM

		Before PSM		Χ^2^/Z	P	After PSM		Χ^2^/Z	*P*
		IR	PR			IR	PR		
IBM	< 25	40 (21.05)	91 (47.89)	3.87	0.049	29 (37.18)	29 (37.18)	0	> 0.05
	> = 25	10 (5.26)	49 (25.79)			10 (12.82)	10 (12.82)		
Sex	Male	32 (16.84)	64 (33.68)	4.93	0.026	21 (26.92)	21 (26.92)	0	> 0.05
	Female	18 (9.47)	76 (40)			18 (23.09)	18 (23.08)		
Smoking	No	45 (23.68)	136 (71.58)	4.17	0.041	39 (50)	37 (47.44)	2.83	0.093
	Yes	5 (2.63)	4 (2.11)			0 (0)	2 (2.56)		
Injured side	Left	30 (15.79)	85 (44.74)	.008	0.93	23 (29.49)	20 (25.64)	0.47	0.49
	Right	20 (10.53)	55 (28.95)			16 (20.51)	19 (24.36)		
Diabetes	No	41 (21.58)	120 (63.16)	.39	0.53	32 (41.03)	31 (39.74)	0.083	0.77
	Yes	9 (4.74)	20 (10.53)			7 (8.97)	8 (10.26)		
Occupation	Non-heavy manual workers	37 (19.47)	109 (57.37)	.31	0.58	32 (41.03)	30 (38.46)	0.32	0.58
	Heavy manual workers	13 (6.84)	31 (16.32)			7 (8.97)	9 (11.54)		
Interval between injury and surgery	< = 72 h	17 (8.95)	56 (29.47)	.56	0.45	11 (14.10)	9 (11.54)	0.27	0.60
	> 72 h	33 (17.37)	84 (44.21)			28 (35.90)	30 (38.46)		
Reduction type	Closed reduction	45 (23.68)	129 (67.89)	0.22	0.64	34 (43.59)	35 (44.87)	.13	0.72
	Open reduction	5 (2.63)	11 (5.79)			5 (6.41)	4 (5.13)		
Fracture site	Subcapital	27 (14.21)	49 (25.79)	8.81	0.012	20 (25.64)	15 (19.23)	8.67	0.013
	Transcervical	16 (8.42)	79 (41.58)			12 (15.38)	23 (29.49)		
	Substrate	7 (3.689)	12 (6.32)			7 (8.97)	1 (1.28)		
Garden Classification	II	0 (0)	31 (16.33)	26.67	0.001	0 (0)	1 (1.28)	1.12	0.57
	III	9 (4.74)	51 (26.85)			9 (11.54)	10 (12.82)		
	IV	41 (21.58)	58 (30.53)			30 (38.46)	28 (35.90)		
Pauwels Classification	I	5 (2.63)	37 (19.47)	8.56	0.014	5 (6.41)	7 (8.97)	.46	0.80
	II	23 (12.11)	67 (35.26)			17 (21.79)	17 (21.79)		
	III	22 (11.58)	36 (18.95)			17 (21.79)	15 (19.23)		
Age (years)		55 (42.75,59.25)	50.5 (38.25,60)	-1.05	0.29	53 (40,59)	54 (38,60)	-0.15	0.88

### Interaction between variables and reoperation after PSM analysis

**Fig. 3:**
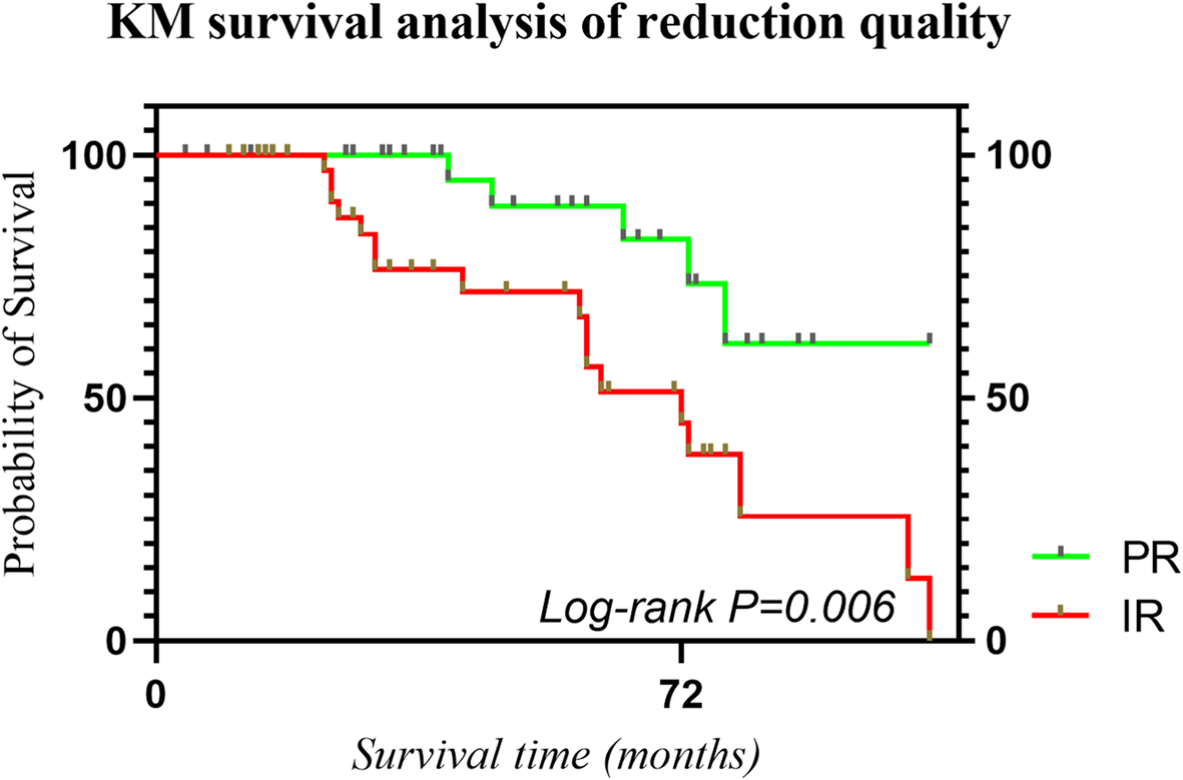
Kaplan–Meier cumulative reoperation rate was higher in Group IR than in Group PR after propensity score-matching analysis (Log-rank *P* = 0.041)

PSM analysis was performed using a multivariable logistic regression model based on: sex, BMI, age, smoking, injured side, diabetes, occupation, interval between the injury and surgery, location of fracture line, Garden classification, Pauwels classification. Pairs of patients receiving perfect reduction (Group PR) and imperfect reduction (Group IR) were derived using 1:1 greedy nearest neighbor matching within one-quarter of the standard deviation of the estimated propensity. This strategy resulted in 39 matched pairs in each group (Table 1). Kaplan–Meier (KM) survival analysis (for the categorical variables) and Cox survival analysis (for the continuous variables)were employed to explore the interaction between each measurement data and reoperation one by one. If the P value was less than 0.1, this group of variables would be included in the subsequent Cox analysis as a suspicious influencing variable of reoperation and a Cox proportional-hazards model was constructed to evaluate the hazard ratio for each event and reoperation as the procedure mentioned in patients after PSM analysis (Table 2). Finally, KM survival analysis of reduction quality was performed as a subgroup analysis (Fig. 3).

**Table 2:** Univariate survival analysis of reoperation before PSM and after PSM

		Before PSM		P	After PSM		*P*
		Reoperation			Reoperation		
		No (%)	Yes (%)		No (%)	Yes (%)	
IBM	< 25	107 (56.32)	24 (12.63)	0.61	44 (56.41)	14 (17.95)	0.17
	> = 25	50 (26.32)	9 (4.74)		12 (15.38)	8 (10.26)	
Sex	Male	80 (42.11)	16 (8.42)	0.80	32 (41.03)	10 (12.82)	0.35
	Female	77 (40.53)	17 (8.95)		24 (30.77)	12 (15.38)	
Smoking	No	149 (78.42)	32 (16.84)	0.61	54 (69.23)	22 (28.21)	0.37
	Yes	8 (4.21)	1 (0.53)		2 (2.56)	0 (0)	
Injuried side	Left	96 (50.53)	19 (10)	0.70	29 (37.18)	14 (17.95)	0.34
	Right	61 (32.11)	14 (7.37)		27 (34.62)	8 (10.26)	
Diabetes	No	132 (69.47)	29 (15.26)	0.58	45 (57.69)	18 (23.08)	0.88
	Yes	25 (13.16)	4 (2.11)		11 (14.1)	4 (5.13)	
Occupation	Non-heavy manual workers	123 (64.74)	23 (12.11)	0.28	46 (58.97)	16 (20.51)	0.35
	Heavy manual workers	34 (17.89)	10 (5.26)		10 (12.82)	6 (7.69)	
Interval between injury and surgery	< = 72 h	61 (32.11)	12 (6.32)	0.79	12 (15.38)	8 (10.26)	0.17
	> 72 h	96 (50.53)	21 (11.05)		44 (56.41)	14 (17.95)	
Reduction type	Closed reduction	145 (76.32)	29 (15.26)	0.40	50 (64.1)	19 (24.36)	0.72
	Open reduction	12 (6.32)	4 (2.11)		6 (7.69)	3 (3.85)	
Reduction quality	Imperfect	30 (15.79)	20 (10.53)	< 0.001	22 (28.21)	17 (21.79)	0.003
	Perfect	127 (66.84)	13 (6.84)		34 (43.59)	5 (6.41)	
Location of fracture line	Subcapital	54 (28.42)	22 (11.58)	0.002	20 (25.64)	15 (19.23)	0.018
	Transcervical	85 (44.74)	10 (5.26)		28 (35.9)	7 (8.97)	
	Basicervical	18 (9.47)	1 (0.53)		8 (10.26)	0 (0)	
Garden Classification	II	30 (15.79)	1 (0.53)	0.001	1 (1.28)	0 (0)	0.30
	III	55 (28.95)	5 (2.63)		16 (20.51)	3 (3.85)	
	IV	72 (37.89)	27 (14.21)		39 (50)	19 (24.36)	
Pauwels Classification	I	35 (18.42)	7 (3.68)	0.99	8 (10.26)	4 (5.13)	0.30
	II	74 (38.95)	16 (8.42)		22 (28.21)	12 (15.38)	
	III	48 (25.26)	10 (5.26)		26 (33.33)	6 (7.69)	
Age (years)(QSD)		53.1 (38.5,59)	57 (41,59.5)	0.011	50.5 (38,58.5)	56.5 (51.5,59.25)	0.068

Results above were considered significant at *P* < 0.05. Statistical analyses were carried out using SPSS V20 (IBM, Armonk, NY, USA) and GraphPad version 8.4.3(686) (GraphPad Software, LLC).

## Results

A total of 190 patients treated between November 2005 and November 2019 were consecutively included in this analysis.

### Interaction between variables and reoperation before PSM analysis

Before PSM analysis, the mean age was 49.96 ± 12.02 years and the total reoperation rate was 17.40%. The baseline variables between perfect reduction and imperfection reduction were not well balanced (showed in Table 1). As shown in Table 2, reduction type (*P* < 0.001), location of fracture line (*P* = 0.002), Garden Classification (*P* = 0.001), age (*P* = 0.011) were potentially interrelated with the need for reoperation. All variables with a *P* < 0.1 in KM analysis were included in the following Cox analysis. After Cox analysis, only reduction quality was interrelated with the need for reoperation (Table 3).

**Table 3:** Cox regression analysis of reoperation before PSM and after PSM

		Before PSM						After PSM					
		B	Ward	*P*	OR	95.0% of OR		B	Ward	*P*	OR	95.0% of OR	
						Lower limit	Upper limit					Lower limit	Upper limit
Reduction quality	Perfect reduction	-1.53	14.84	< 0.001	0.22	0.1	0.47	-1.90	10.57	0.001	0.15	0.048	0.47
	Imperfect reduction				1			0			1		
Fracture site	Transcervical	-0.25	0.35	0.55	0.78	0.35	1.75	0.83	2.08	0.15	2.29	0.74	7.08
	Substrate	-1.58	2.35	0.13	0.21	0.028	1.55	-13.88	0.001	0.98	0	0	0
	Subcapital	0			1			0			1		
Garden Classification	III	0.51	0.21	0.65	1.67	0.19	15.07	-	-	-	-	-	-
	IV	1.50	2.02	0.16	4.47	0.57	35.29	-	-	-	-	-	-
	II	0			1			-	-	-	-	-	-
Age		0.032	3.81	0.051	1.03	1	1.07	0.041	3.7	0.054	1.04	0.99	1.09

### Interaction between the quality of the reduction and reoperation after PSM analysis

After 1:1 propensity score matching (PSM), the baseline variables between the two groups were well balanced (Table 1). The mean age was 49.37 ± 12.36 and the total reoperation rate was 28.2%. The median duration of follow-up for Group PR and Group IR was 43.69 ± 26.06 and 43.43 ± 29.06 months. Univariate survival analysis showed that reduction quality (*P* = 0.003), location of fracture line (*P* = 0.018) and age (P-0.068) were potentially interrelated with cumulative reoperation rate (Table 2). Cox analysis showed that only reduction quality was interrelated with the need for reoperation after PSM analysis (Table 3). As a subgroup analysis (Fig. 3), Kaplan–Meier cumulative reoperation rate was higher in Group IR than in Group PR after propensity score-matching analysis (Log-rank *P* = 0.041).

## Discussion

In the past 30 years, the treatment of femoral neck fractures is becoming increasingly standardized with the development of better internal fixation materials and improvements in surgical technique, but the reoperation rate has not improved significantly [1, 11]. Femoral head necrosis and fracture nonunion have always been the two major complications of femoral neck fracture treatment, which greatly increases the difficulty of treatment and places a high burden on social and medical resources [12, 13]. The final treatment outcome of these two complications is conversion surgery to a revision or hip replacement surgery (such as hemiarthroplasty or total hip arthroplasty). Even experienced surgeons are still uncertain about the iatrogenic risk factors affecting the need for a reoperation [14–16]. Therefore, this study aims to analyze the risk factors related to reoperation and provides theoretical references for future clinical research. The evidence derived from randomized controlled studies is certainly the most solid, but femoral neck fractures are mostly caused by accidental injuries, and their incidence is not high enough to easily carry out randomized controlled studies. Therefore, we established clear inclusion and exclusion criteria to carry out a retrospective study with PSM analysis, which has clinical significance second only to randomized controlled studies.

As it’s known to all, compared with the clearer diagnostic criteria of femoral head necrosis, nonunion is often missed or not diagnosed in a timely fashion [17–19]. Early and mid-term femoral head necrosis does not completely affect the use of the patient’s hip joint. For young patients, delaying the time of hip replacement as long as possible is an effective treatment strategy on the premise of meeting the needs of the patient’s hip joint. The occurrence of femoral necrosis thus does not completely represent failure of fixation of a femoral neck fracture [2, 20]. The X-ray signs of early femoral head necrosis are not obvious. Therefore, our study adopted a reoperation for a hip fracture (a revision or hemiarthroplasty or total hip arthroplasty),which represents a failure of treatment, as the ending point of primary femoral neck fracture treatment. Cox multivariate survival analysis was used to analyze the survival time from primary treatment to reoperation. Before PSM analysis, only the reduction quality affected the cumulative effect of femoral neck fracture that requires reoperation (Table 3). After finding the unique influence of anatomic reduction and reduction quality maintenance on the treatment of femoral neck fracture, we started to consider dividing the patients into perfect reduction and imperfect reduction, matched them with PSM, so as to balance the baseline influencing factors between the two groups and make the results of survival analysis more reliable. The results of multivariate survival analysis before and after PSM analysis showed that only the reduction quality was related to the cumulative risk of reoperation for femoral neck fracture. We are more convinced that what doctors can do with the non-elderly with femoral neck fracture is to achieve as perfect reduction as possible and maintain the stability of reduction until the patient fully recovers. Open reduction and more stable internal fixation instruments should be used if necessary. Previous studies have confirmed that the key factor affecting the final treatment outcome of patients is not the reduction method and surgical approach [21–23]. Even open reduction will not affect the final treatment outcome of patients, because the correct approach and gentle operation will not affect the blood supply of patients' femoral head in theory. In this study, the median survival time of femoral head with imperfect reduction was 72 months, and the survival rate of femoral head with perfect reduction was still more than 80% in 72th month (Fig. 3).

Orthopedic experts believe that the magnitude of shear force, the interval between injury and primary operation, the position of fracture line and the quality of fracture reduction are major important factors affecting the prognosis of femoral neck fracture [24]. This study only concluded that the quality of reduction is the only factor affecting the reoperation rate of femoral neck fracture. Varus deformity increases the shear stress, while valgus deformity can reduce the shear stress. Therefore, in clinical practice, if complete anatomic reduction cannot be achieved, the angle requirements for valgus are more extensive than those for varus deformity. Even so, excessive valgus may not yield good results because the artery supplying the femoral head may be distorted and the reduction of valgus may not effectively restore blood supply [25]. After discussion, our team agreed that this cannot overturn the understanding of orthopedics doctors. There are two possible reasons: (1) reoperation rate was a single index to evaluate the related risk factors femoral neck fracture. In principle, we could increase the evaluation indexes, such as the rate of femoral head necrosis and the rate of fracture nonunion. However, these two indexes required patients to return to the hospital for reexamination as soon as symptoms appear. The reality is that many patients with early avascular necrosis of the femoral head do not have any symptoms. They cannot return to the hospital on time for review; (2) the small sample size is not enough to find the influence of fracture line position, fracture block displacement size, shear force size and the interval between injury and surgery. Even so, it does not affect the practical guiding significance of this study, because among the five influencing factors generally considered (the influence of fracture line position, fracture block displacement size, the interval between injury and surgery, shear force size and reduction quality), only reduction quality are iatrogenic influencing factors. Although it's a bit trite, the rapid development of medical technology and materials does not affect the treatment principle of fractures. In clinical research, as for non-elderly femoral neck fractures, orthopedics doctors should be bolder in achieving anatomical reduction and obtaining reliable fixation.

## Conclusion

To prolong the service life of the original femoral head, it is essential to achieve a completely anatomical reduction and maintain the reduction quality until the patient fully recovers.

## Data Availability

All data generated or analysed during this study are included in this article.

## Abbreviations

CRIF: Closed reduction and internal fixation
ORIF: Open reduction and internal fixation
DHS: Dynamic hip screw
PSM: Propensity score matching analysis
HA: Hemiarthroplasty
THA: Total hip arthroplasty
KM analysis: Kaplan–Meier analysis
